# Supplementary material for: Uncovering the chiral bias of meteoritic isovaline through asymmetric photochemistry
Source: Nat Commun. 2023 Jun 8;14:3381. doi: 10.1038/s41467-023-39177-y (PMC10250315; doi:10.1038/s41467-023-39177-y)
Supplement: Supplementary file 1 — Supplementary information [file 41467_2023_39177_MOESM1_ESM.pdf]

SUPPLEMENTARY INFORMATION

Uncovering the chiral bias of meteoritic isovaline through asymmetric photochemistry

Jana Bocková[a], Nykola C. Jones[b], Jérémie Topin[a], Søren V. Hoffmann[b] and Cornelia Meinert[a*]

[*]*Corresponding author's e-mail: cornelia.meinert@univ-cotedazur.fr*

This file contains details of the circular dichroism/anisotropy spectroscopy data treatment, comparison of the anisotropy spectra with previous data, analysis of scanning electron microscopy images, complementary information on multidimensional gas-chromatography coupled to reflectron time-of-flight mass-spectrometry analyses, quantum chemical calculations results, and a list of isovaline enantiomeric excesses detected in carbonaceous chondrites.

It contains Supplementary Figs. 1–5, and Supplementary Tables 1–3.

Supplementary Notes

Supplementary Note 1: Kagan's equation[1] **1**

Supplementary Note 2: VUV/UV anisotropy spectroscopy of isotropic isovaline films **2**

Absorption, circular dichroism and anisotropy data 2

Comparison of the present anisotropy spectra with Meinert *et al.* (2012)[2] 3

Scanning electron microscopy (SEM) imaging 5

Supplementary Note 3: Quantum chemical calculations **6**

Supplementary Note 4: Multidimensional gas chromatographic analysis of isovaline residues **7**

Supplementary Note 5: Enantiomeric excesses of isovaline detected in carbonaceous chondrites **8**

Supplementary References 9

Supplementary Note 1: Kagan's equation[1]

Kagan's equation[1] describes how the enantiomeric excess ee_{L} of a racemic mixture in an asymmetric photolysis evolves with the extent of reaction ξ:

$$\xi = 1 - \frac{1}{2}\left[\left(\frac{1+ee_{\mathrm{L}}}{1-ee_{\mathrm{L}}}\right)^{\frac{1}{2}-\frac{1}{g_{\mathrm{L}}}} + \left(\frac{1+ee_{\mathrm{L}}}{1-ee_{\mathrm{L}}}\right)^{-\frac{1}{2}-\frac{1}{g_{\mathrm{L}}}}\right] \quad (1)$$

where g_{L} is the anisotropy factor corresponding to the L-enantiomer and depends on the wavelength of the circularly polarized light.

Supplementary Note 2: VUV/UV anisotropy spectroscopy of isotropic isovaline films

Absorption, circular dichroism and anisotropy data treatment

The measurements of three independent thin films were chosen for the final data set for each of the two enantiomers based on their low level of scattering. Each set of measurements is represented by a thin dashed line in Supplementary Fig. 1a and b. These thin lines are averages of the absorption and CD spectra recorded for four different angles (0°, 90°, 180°, and 270°) defined by rotating the window holder around the axis of the incident synchrotron radiation normal to the sample surface. The level of agreement between the absorption and CD spectra recorded at these four angles for each sample allows us to exclude any significant effects due to linear birefringence and/or linear dichroism[3,4]. For better comparison between individual films of varying thicknesses, a normalization was applied to the CD spectra by scaling their corresponding absorbance in the wavelength range 155–180 nm. The final normalized absorption spectra are in Supplementary Fig. 1c and the normalized CD spectra are in Supplementary Fig. 1d. Note that the here applied normalization process has no influence on the anisotropy spectra, $g = \Delta\varepsilon/\varepsilon$ = *CD / absorbance*, as they are independent of any scaling factor applied to the absorbance and CD spectra. The resultant anisotropy spectra for both isovaline enantiomers are presented in Supplementary Fig. 2.

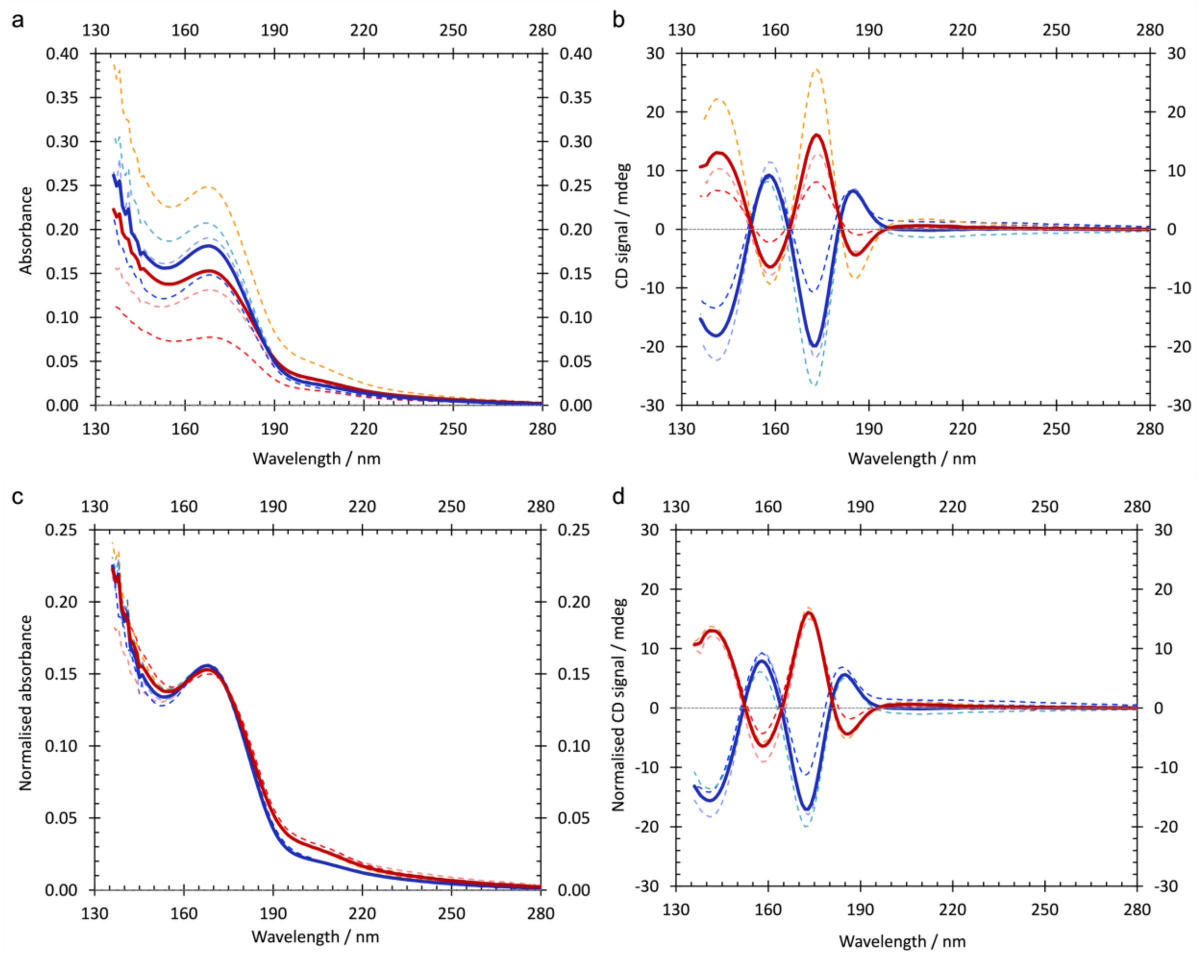

Supplementary Fig. 1 Synchrotron radiation absorption and CD data. Absorbance and CD spectra of the L- (blue shading) and D- (red shading) enantiomers of isovaline: **a** and **b** before, and **c** and **d** after normalization, respectively. Thin dashed lines represent averaged data recorded at four different angles (0°, 90°, 180°, and 270°) for each film. Thick lines represent average over 3 films for the L- (blue) and D- (red) enantiomers. Source data are provided as a Source Data file.

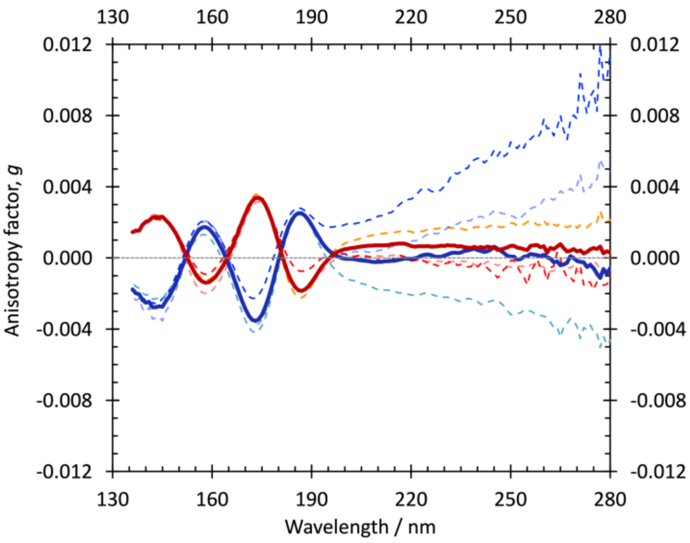

Supplementary Fig. 2 Anisotropy spectra of the L- (blue shading) and D- (red shading) enantiomers of isovaline. Thin dashed lines represent averaged data recorded at four different angles (0°, 90°, 180°, and 270°) for each film. Thick lines represent average over 3 films for the L- (blue) and D- (red) enantiomers.

Comparison of the present anisotropy spectra with previous data

The level of scattering we reached in the CD/anisotropy spectroscopy experiments on solid-phase L- and D-isovaline with the present sample preparation procedure was significantly reduced compared to our previous attempts of producing thin films of isovaline enantiomers using sublimation followed by deposition in a high vacuum sublimation-deposition chamber (Meinert et al., 2012[2]). This is manifested by well-defined and quasi-perfectly mirrored anisotropy bands and zero-crossings of the two enantiomers in the present study (Supplementary Fig. 3a) as opposed to 2012[2]. Even though the overall anisotropy signal (zero crossings, band signs, shapes, and intensities) from 2012 is significantly distorted, the green and purple bars in Supplementary Fig. 3a highlight the general agreement in the positions of the anisotropy bands in between the two data sets.

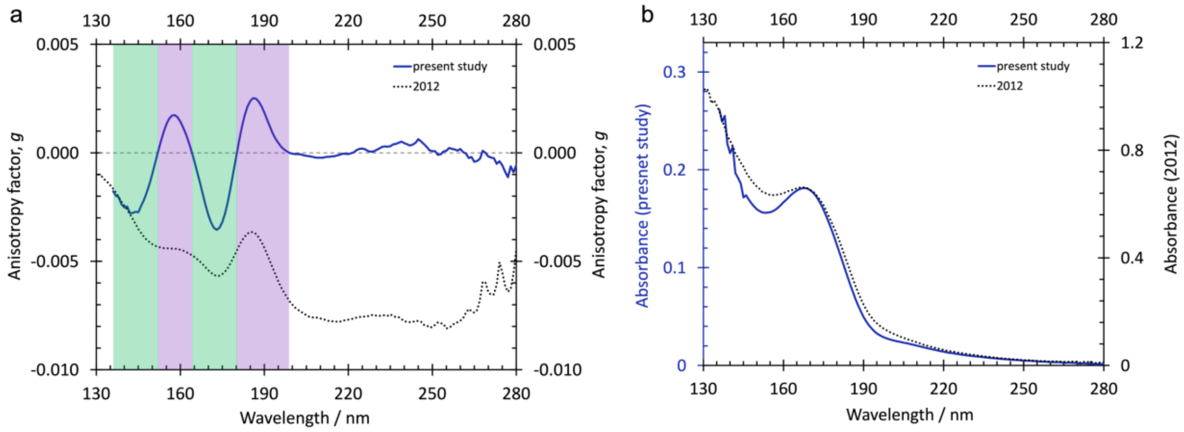

Supplementary Fig. 3 Comparison of (a) anisotropy and (b) absorbance spectra of L-isovaline from the present study and Meinert et al. (2012)[2]. a There is a general agreement in the positions of the anisotropy bands of L-isovaline between the present study (blue line) and the previous study in 2012[2] (dotted line), while the band shape, signs, intensities, and zero crossings in the 2012 anisotropy spectra are clearly distorted by scattering effects. **b** The main features in the absorbance spectra of L-isovaline are consistent in both the present study and the previous study in 2012[2].

To elucidate the effect of the sample preparation procedure (drop-casting *vs* sublimation-deposition) on sub-/microscopic film properties and hence their spectroscopic response, we performed scanning electron microscopy (SEM) imaging (Supplementary Information, S.1.3). Even though the exact same films could not be used for both SEM and CD/anisotropy spectroscopy, the selection of films for the SEM imaging was based on the similarity of their macroscopic appearance with the ones prepared in analogous conditions for the spectroscopy experiments. The L-isovaline film in Supplementary Fig. 4a–c prepared by drop-casting is mostly amorphous with only sporadic nanocrystal nucleation sites, the presence of which could potentially explain the minor distortions in the CD and hence anisotropy spectra in the present study. Such nanocrystal nucleation sites are much more abundant in the ~200 nm L-isovaline film prepared by sublimation-deposition (Supplementary Fig. 4d–f) and turn to plate submicrocrystals with increasing film thickness (Supplementary Fig. 4g–i). This is likely to explain the artefacts in the 2012 anisotropy spectrum in Supplementary Fig. 3a, where based on the absorbance (Supplementary Fig. 3b), the film thickness was significantly larger compared to the present study. It is likely that the slow deposition of enantiopure gas-phase isovaline at elevated temperatures as opposed to fast methanol evaporation at moderate temperatures during drop-casting favours sub-/microcrystal growth. Clearly, the suite of isovaline structures and their relative abundance differs in the gas-phase (neutrals) and methanol solution (zwitterions). It is therefore possible that the former one exhibits higher predisposition to forming crystallisation nucleation sites, which are known to be able to further affect the conformations of crystallising species and hence facilitate the crystal growth[5]. On the contrary, the ~400 nm film of racemic isovaline in Supplementary Fig. 4j–l exhibits non-crystalline isotropic amorphous character with no long-range order.

Scanning electron microscopy (SEM) imaging

SEM images of L- and DL-isovaline thin films prepared by drop-casting and/or sublimation-deposition techniques (Supplementary Fig. 4) were recorded using JEOL JSM-6700F scanning electron microscope at Microscopie Imagerie Côte d'Azur. Prior SEM imaging, the CaF_2 windows with the isovaline films were fixed using silver conductive paste to metallic support plates and coated with a 2 nm thick platinum layer.

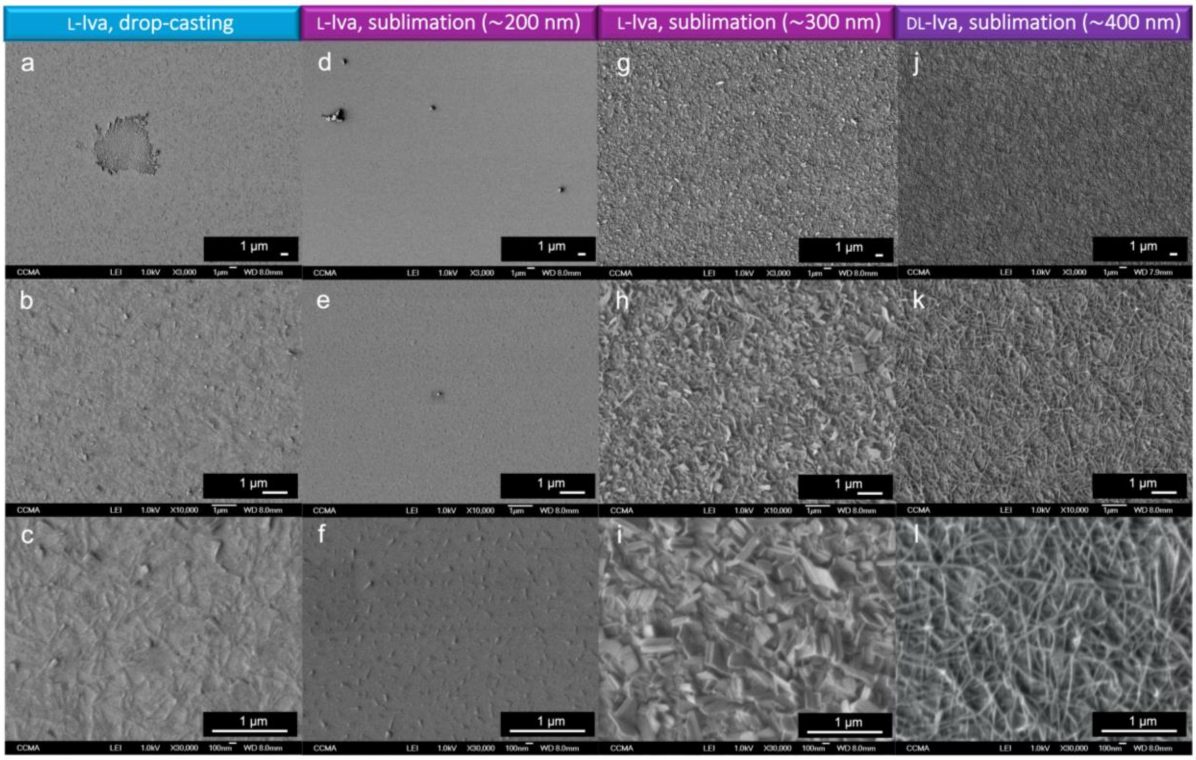

Supplementary Fig. 4 Distinct morphology of the L- and DL-isovaline films prepared by drop-casting and/or sublimation-deposition techniques. a–c SEM images of L-Isovaline film prepared by evaporation of 60 µL of 1 mg mL^{-1} L-isovaline standard solution in methanol at three different scales. A growing fan-like microcrystal in **a** surrounded by large amorphous areas with dispersed scarce nanocrystal nucleation sites are clearly visible in **b–c**. **d–f** An ~200 nm thick L-isovaline film prepared by sublimation of L-isovaline at 120 °C followed by deposition on a CaF_2 window in the sublimation-deposition chamber (pressure ~10^{-5} mbar). The well distinguished grains in **d** represent unwanted dust particles fallen on the surface of the film. Numerous nanocrystal nucleation sites are visible in **e–f**. **g–i** An ~300 nm thick L-isovaline film prepared by sublimation of L-isovaline at 133 °C followed by deposition on a CaF_2 window in the sublimation-deposition chamber (pressure ~10^{-5} mbar) dominated by plate submicrocrystals. **j–l** The amorphous ~400 nm thick racemic DL-isovaline film prepared by sublimation of DL-isovaline standard at 115 °C followed by deposition on a CaF_2 window in the sublimation-deposition chamber (pressure ~10^{-7} mbar) is comprised of nanofiber networks exhibiting no long-range order. The white bars in the bottom right corners of the SEM images indicate a length of 1 µm.

Supplementary Note 3: Quantum chemical calculations

The initial molecular structures of isovaline were built using GaussView (version 6)[5]. Four distinct conformers **Ia**, **IIa**, **IIb**, and **IIc** were taken from previous work[6]. After converting each neutral structure to the zwitterionic form, theoretical calculations were carried out using time-dependent density functional theory (TDDFT) with the B3P86 hybrid functional in combination with the 6-311+G(d,p) basis set. In total, 200 excited states were calculated for each of the four conformers. The theoretical electronic circular dichroism spectra have been convoluted by means of summing rotatory strength weighted Gaussian distribution functions with full width at half maximum of 0.33 eV. All calculations were made using Gaussian 16 Rev. C.01.[7].

Among the four considered conformers, only conformer **IIa** provided a good agreement with the experimental CD spectrum (Supplementary Fig. 5). The energetically favored configuration according to the TDDFT calculation did not correlate most strongly with the experiment which is consistent with previous observations for isotropic films of alanine[8].

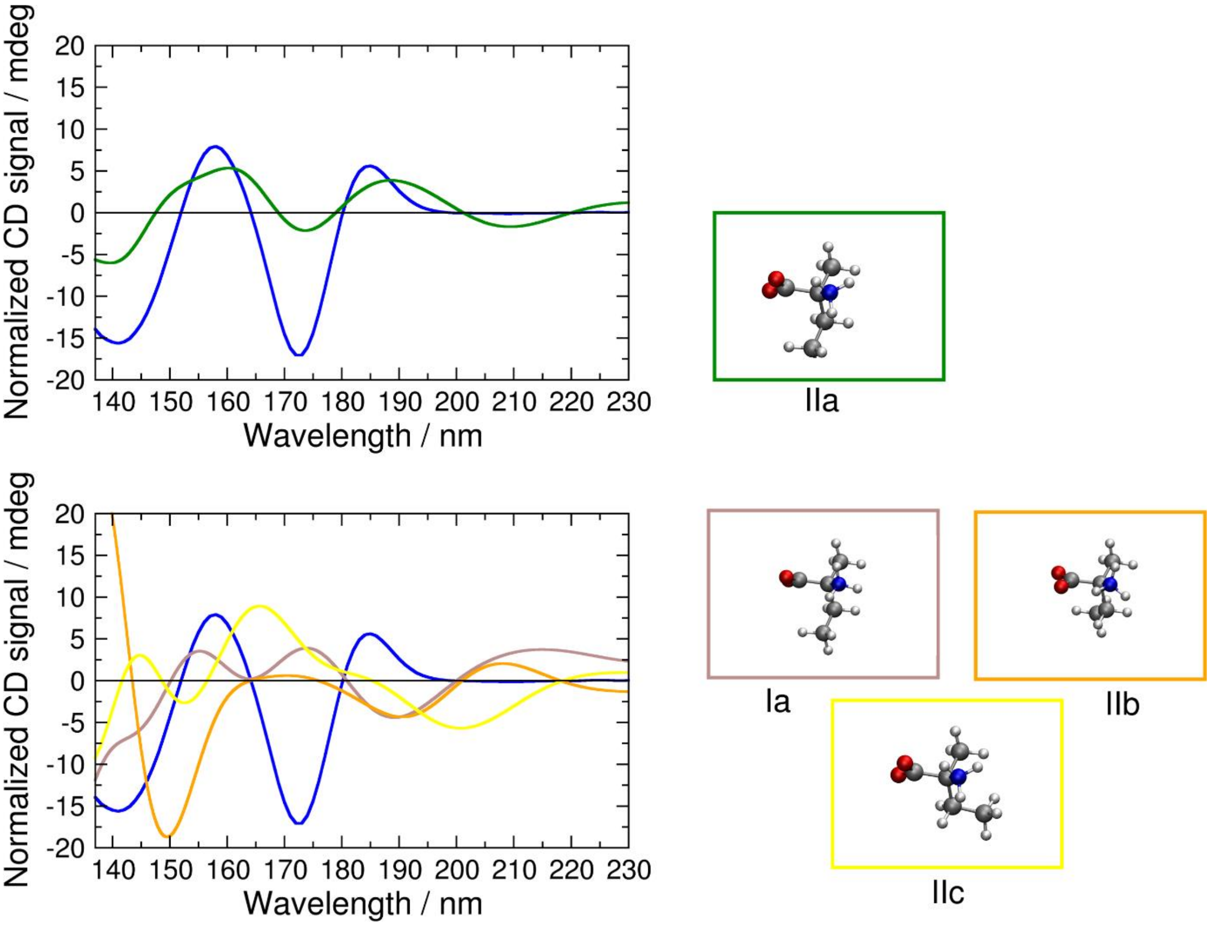

Supplementary Fig. 5 Comparison of the calculated and experimental CD spectra. The blue line accounts for the experimental CD spectrum of L-isovaline. The theoretical spectra were obtained using time-dependent density functional theory with the B3P86 hybrid functional in combination with the 6-311g+(d,p) basis set. Among the four most stable conformers, only **IIa** agrees qualitatively with the experimental spectrum (upper panel, green line). The lower panel displays the theoretical spectra obtained for the three conformers **Ia**, **IIb**, and **IIc** as brown, orange, and yellow line, respectively.

Supplementary Note 4: Multidimensional gas chromatographic analysis of isovaline residues

Supplementary Table 1 Enantiomeric excess values measured by GC×GC–TOF-MS of the irradiated ($\%ee_{L\_irr}$) and the corresponding non-irradiated isovaline film ($\%ee_{L\_non\text{-}irr}$) along with the t-values and degrees of freedom for the Student's t-Tests confirming the statistical significance of calculated *ee*-s reported in Table 1.

Sample set	CPL helicity	$\%ee_{L\_irr} \pm SD_{irr}$ (n_{irr})	$\%ee_{L\_non\text{-}irr} \pm SD_{non\text{-}irr}$ ($n_{non\text{-}irr}$)	Two-sample t-Test (two-tailed), t-value (degrees of freedom)
I	L-CPL	2.83 ± 0.30 (9)	4.89 ± 1.01 (16)	2.07 (23)
II	L-CPL	−0.87 ± 0.81 (9)	0.15 ± 0.26 (9)	2.12 (16)
III	r-CPL	4.59 ± 0.92 (9)	2.71 ± 0.55 (10)	2.11 (17)
IV	r-CPL	3.83 ± 0.43 (9)	2.56 ± 0.28 (9)	2.12 (16)

Supplementary Table 2 Enantiomeric excesses of a set of racemic diluted (1:2000), non-irradiated DL-isovaline films are the same within statistical uncertainties.

Sample	$\%ee_L \pm SD$	Number of replicate GC×GC injections	two-sample t-Test, p-value (two-tailed)	two-sample t-Test (two-tailed), t-value (degrees of freedom)
a	1.35 ± 0.58	6	0.51	2.22 (10)
b	1.54 ± 0.34	6		

Supplementary Note 5: Enantiomeric excesses of isovaline detected in carbonaceous chondrites

Supplementary Table 3 Enantiomeric excesses of L-isovaline (*ee_L*) detected in carbonaceous chondrites. The detections with shading in the "%ee_L ± SD ($n_{injections}$)" column are plotted in **Fig. 4** in the main manuscript, with the colour of the shading in the table relating to the colour of the symbols used in **Fig. 4**.

Name of chondrite	Type of chondrite	Petrologic class	%ee_L ± SD ($n_{injections}$)	Reference
Orgueil	CI	1.1	17.5 ± 30.7 (4)	Martins, 2007[a]
Orgueil	CI	1.1	15.2 ± 4 (8)	Glavin, 2009[6]
Ivuna	CI	1.1	6.6 ± 3.9 (4)	Burton, 2014[7]
Tagish Lake 5b	CI/CM	1.9	7.0 ± 1.9 (8)	Glavin, 2012[8]
Murchison	CM	1.6	8.4 ± 0.8 (8)	Cronin, 1997[9]
			13 ± 30 (2)	Botta, 2002[b]
			3.6 ± 0.3 (7)	Pizzarello, 2003[10]
			3 ± 1.3 (5)	
			6 ± 0.4 (5)	
			5.2 ± 0.5 (14)	
			15.2 ± 0.2 (8)	
			12.6 ± 0.4 (6)	
			0.2 ± 0.3 (6)	
			3.4 ± 0.6 (8)	
			10.6 ± 0.6 (33)	
			-4.1 ± 12.9 (4)	Martins, 2007[a]
			18.5 ± 2.6 (20)	Glavin, 2009[6]
			17.2 ± 6.7 (8)	Glavin, 2010[11]
			9.7 ± 1.2 (3)	Aponte, 2014[c]
			7.2 ± 9.3 (3)	Burton, 2014[d]
			5.6 ± *NR* (*NR*)	Callahan, 2014[12]
			4.61 ± 0.28 (5)	Myrgorodska, 2016[13]
			6.1 ± 5 (4)	Friedrich, 2019[e]
			10 ± 1 (6)	Glavin, 2020[14]
Murray	CM	1.5	6 ± 0.5 (20)	Pizzarello, 2000[15]
Mighei	CM	1.6	8 ± 43 (2)	Botta, 2002[b]
LEW 90500	CM	1.6	3.3 ± 1.8 (23)	Glavin, 2009[6]
LON 94102	CM	1.8	2.4 ± 4.1 (8)	
SCO 06043	CM	1.2	16.5 ± 7.5 (8)	Glavin, 2010[11]
Paris	CM	2.7	-1.4 ± 2.6 (9)	Martins, 2015[16]
Mukundpura	CM	1	9.7 ± *NR* (*NR*)	Pizzarello, 2018[17]
Aguas Zarcas	CM	2	15 ± 7 (6)	Glavin, 2020[14]
			11 ± 6 (6)	

Supplementary Table 3 *to be continued.*

Name of chondrite	Type of chondrite	Petrologic class	*%ee*$_L$ ± SD ($n_{injections}$)	Reference
Asuka 12236	CM	3.0	-2.4 ± 4.5 (6)	Glavin, 2020[18]
GRA 95229	CR	2.5	3 ± *NR* (*NR*)	Pizzarello, 2008[19]
EET 92042	CR	2.5	-1 ± 4.3 (8)	Glavin, 2009[6]
QUE 99177	CR	2.4	0.3 ± 2.1 (8)	
GRO 95577	CR	1.3	11.0 ± 7.2 (8)	Glavin, 2010[11]
MIL 090001	CR	2.4	10 ± 10 (5)	Aponte, 2020[20]
MIL 05082	CB	*NR*	9.7 ± 3 (6)	Burton, 2013[21]
MIL 07411	CB	*NR*	14 ± 4 (6)	
ALH 85085	CH	3	20.5 ± 7.1 (6)	
PCA 91467	CH	3	13 ± 3 (6)	
PAT 91546	CH	3	5 ± 2 (6)	
Tagish Lake 11h	$C_{ungrouped}$	*NR*	0 ± 2.8 (8)	Glavin, 2012[8]
Tagish Lake 1	$C_{ungrouped}$	*NR*	6.7 ± 5.9 (3)	Simkus, 2019[f]
Tagish Lake 10a	$C_{ungrouped}$	*NR*	0 ± 4.1 (3)	

NR – not reported.
[a]Reported by Glavin *et al.*[18] with reference to Martins *et al.*[22]
[b]Reported by Glavin *et al.*[18] with reference to Botta *et al.*[23]
[c]Reported by Glavin *et al.*[18] with reference to Aponte *et al.*[24]
[d]Reported by Glavin *et al.*[18] with reference to Burton *et al.*[25]
[e]Reported by Glavin *et al.*[18] with reference to Friedrich *et al.*[26]
[f]Reported by Glavin *et al.*[18] with reference to Simkus *et al.*[27]

Supplementary References

1. Balavoine, G., Moradpour, A. & Kagan, H. B. Preparation of chiral compounds with high optical purity by irradiation with circularly polarized light, a model reaction for the prebiotic generation of optical activity. *J. Am. Chem. Soc.* **96**, 5152–5158 (1974).
2. Meinert, C. *et al.* Anisotropy spectra of amino acids. *Angew. Chemie Int. Ed.* **51**, 4484–4487 (2012).
3. Kuroda, R. Circular dichroism in the solid state. *Chiral Photochemistry*, 385–414 (CRC Press, 2004).
4. Lees, J. G., Smith, B. R., Wien, F., Miles, A. J. & Wallace, B. A. CDtool - An integrated software package for circular dichroism spectroscopic data processing, analysis, and archiving. *Anal. Biochem.* **332**, 285–289 (2004).
5. Derdour, L. & Skliar, D. Crystallization from solutions containing multiple conformers. 1. Modeling of crystal growth and supersaturation. *Cryst. Growth Des.* **12**, 5180–5187 (2012).
6. Glavin, D. P. & Dworkin, J. P. Enrichment of the amino acid L-isovaline by aqueous alteration on CI and CM meteorite parent bodies. *Proc. Natl. Acad. Sci. U. S. A.* **106**, 5487–5492 (2009).
7. Burton, A. S., Grunsfeld, S., Elsila, J. E., Glavin, D. P. & Dworkin, J. P. The effects of parent-body hydrothermal heating on amino acid abundances in CI-like chondrites. *Polar Sci.* **8**, 255–263 (2014).
8. Glavin, D. P. *et al.* Unusual nonterrestrial L-proteinogenic amino acid excesses in the Tagish Lake meteorite. *Meteorit. Planet. Sci.* **47**, 1347–1364 (2012).

9. Cronin, J. R. & Pizzarello, S. Enantiomeric excesses in meteoritic amino acids. *Science.* **275**, 951–955 (1997).
10. Pizzarello, S., Zolensky, M. & Turk, K. A. Nonracemic isovaline in the Murchison meteorite: Chiral distribution and mineral association. *Geochim. Cosmochim. Acta* **67**, 1589–1595 (2003).
11. Glavin, D. P., Callahan, M. P., Dworkin, J. P. & Elsila, J. E. The effects of parent body processes on amino acids in carbonaceous chondrites. *Meteorit. Planet. Sci.* **45**, 1948–1972 (2010).
12. Callahan, M. P., Martin, M. G., Burton, A. S., Glavin, D. P. & Dworkin, J. P. Amino acid analysis in micrograms of meteorite sample by nanoliquid chromatography-high-resolution mass spectrometry. *J. Chromatogr. A* **1332**, 30–34 (2014).
13. Myrgorodska, I., Meinert, C., Martins, Z., d'Hendecourt, L. L. S. & Meierhenrich, U. J. Quantitative enantioseparation of amino acids by comprehensive two-dimensional gas chromatography applied to non-terrestrial samples. *J. Chromatogr. A* **1433**, 131–136 (2016).
14. Glavin, D. P. *et al.* Extraterrestrial amino acids and L-enantiomeric excesses in the CM2 carbonaceous chondrites Aguas Zarcas and Murchison. *Meteorit. Planet. Sci.* **56**, 148–173 (2020).
15. Pizzarello, S. & Cronin, J. R. Non-racemic amino acids in the Murray and Murchison meteorites. *Geochim. Cosmochim. Acta* **64**, 329–338 (2000).
16. Martins, Z., Modica, P., Zanda, B. & D'Hendecourt, L. L. S. The amino acid and hydrocarbon contents of the Paris meteorite: Insights into the most primitive CM chondrite. *Meteorit. Planet. Sci.* **50**, 926–943 (2015).
17. Pizzarello, S. & Yarnes, C. T. The soluble organic compounds of the Mukundpura meteorite: A new CM chondrite fall. *Planet. Space Sci.* **164**, 127–131 (2018).
18. Glavin, D. P. *et al.* Abundant extraterrestrial amino acids in the primitive CM carbonaceous chondrite Asuka 12236. *Meteorit. Planet. Sci.* **55**, 1979–2006 (2020).
19. Pizzarello, S., Huang, Y. & Alexandre, M. R. Molecular asymmetry in extraterrestrial chemistry: Insights from a pristine meteorite. *Proc. Natl. Acad. Sci. U. S. A.* **105**, 3700–3704 (2008).
20. Aponte, J. C. *et al.* Analysis of amino acids, hydroxy acids, and amines in CR chondrites. *Meteorit. Planet. Sci.* **55**, 2422–2439 (2020).
21. Burton, A. S., Elsila, J. E., Hein, J. E., Glavin, D. P. & Dworkin, J. P. Extraterrestrial amino acids identified in metal-rich CH and CB carbonaceous chondrites from Antarctica. *Meteorit. Planet. Sci.* **48**, 390–402 (2013).
22. Martins, Z. *et al.* Amino acid composition, petrology, geochemistry, ^{14}C terrestrial age and oxygen isotopes of the Shişr 033 CR chondrite. *Meteorit. Planet. Sci.* **42**, 1581–1595 (2007).
23. Botta, O., Glavin, D. P., Kminek, G. & Bada, J. L. Relative amino acid concentrations as a signature for parent body processes of carbonaceous chondrites. *Orig. Life Evol. Biosph.* **32**, 143–163 (2002).
24. Aponte, J. C., Dworkin, J. P. & Elsila, J. E. Assessing the origins of aliphatic amines in the Murchison meteorite from their compound-specific carbon isotopic ratios and enantiomeric composition. *Geochim. Cosmochim. Acta* **141**, 331–345 (2014).
25. Burton, A. S. *et al.* The amino acid composition of the Sutter's Mill CM2 carbonaceous chondrite. *Meteorit. Planet. Sci.* **49**, 2074–2086 (2014).
26. Friedrich, J. M. *et al.* Effect of polychromatic X-ray microtomography imaging on the amino acid content of the Murchison CM chondrite. *Meteorit. Planet. Sci.* **54**, 220–228 (2019).
27. Simkus, D. N. *et al.* New insights into the heterogeneity of the Tagish Lake meteorite: Soluble organic compositions of variously altered specimens. *Meteorit. Planet. Sci.* **54**, 1283–1302 (2019).
