# Supplementary material for: Uncovering the chiral bias of meteoritic isovaline through asymmetric photochemistry
Source: Nat Commun. 2023 Jun 8;14:3381. doi: 10.1038/s41467-023-39177-y (PMC10250315; doi:10.1038/s41467-023-39177-y)
Supplement: Supplementary file 3 — Description of Additional Supplementary Files [file 41467_2023_39177_MOESM3_ESM.pdf]

Description of Additional Supplementary Files

File Name: Supplementary Data 1
Description: Equilibrium structures of L-isovaline conformers optimized with Gaussian 16 Rev. C.01. using the B3P86 hybrid functional with the 6-311+G(d,p) basis set. Cartesian coordinates (x, y, z) are given in Å. Excel spreadsheet.
